# On Thermal Insulation Properties of Various Foaming Materials Modified Fly Ash Based Geopolymers

**DOI:** 10.3390/polym15153254

**Published:** 2023-07-30

**Authors:** Yukun Ji, Quanming Ren, Xiaozhao Li, Peng Zhao, Veerle Vandeginste

**Affiliations:** 1 State Key Laboratory for Geomechanics and Deep Underground Engineering, China University of Mining and Technology, Xuzhou 221116, China; yukun.ji@cumt.edu.cn (Y.J.);; 2 Yunlong Lake Laboratory of Deep Earth Science and Engineering, Xuzhou 221116, China; 3 School of Earth Sciences and Engineering, Nanjing University, Nanjing 210023, China; 4 Department Materials Engineering, KU Leuven Campus Bruges, B-8200 Bruges, Belgium

**Keywords:** phenolic resin, hydrogen peroxide, silica aerogel, fly ash geopolymer, thermal insulation performance

## Abstract

Geopolymers can be used as a thermally insulated material because of their considerable porosity, whereas the combined effect of various modifying agents on their heat-insulating properties remains unexplored. Here, orthogonal experiments were carried out to evaluate the thermal insulation performance of fly ash geopolymer modified by phenolic resin, silica aerogel, and hydrogen peroxide. Moreover, variance analysis and range analysis were applied to estimate the influence of modifying agents on the thermal insulation performance of the geopolymer. The results demonstrate that the thermal conductivity of fly ash geopolymer significantly reduces (from 0.48 W/m·K to 0.12 W/m·K) due to the combined effect of the three modifying agents. Based on the variance analysis and range analysis, the optimum thermal conductivity ultimately reaches 0.08 W/m·K via a best composition scheme of the three modifying agents. Moreover, phenolic resin can facilitate the formation of a network structure and increase the porosity of micron pores (>1 μm). Hydrogen peroxide can be decomposed into O_2_ in an alkaline environment and leave large-diameter pores (>1 μm) during curing. Some silica aerogel is embedded in the geopolymer matrix as microspheres with extremely low thermal conductivity, whereas the rest of the silica aerogel may react with the alkali activator to form water, and subsequently leaves pores (>1 μm) after evaporation of water during the curing. In addition, a newly modified Maxwell–Euchen model using iterative calculation and considering the Knudsen effect (pores of micron or even nanometer scale) is proposed and validated by the experimental data. The foamed geopolymer in this research can be used as a reference for building insulation layer design. This research unravels phenolic resin-, silica aerogel-, and hydrogen peroxide-influenced thermal insulation mechanisms of geopolymer that may have impacts on deployment of a thermally insulating material in the construction field.

## 1. Introduction

With the increasingly prominent global energy problem and the high carbon emissions related to the production of ordinary Portland cement (OPC), acceptable alternatives to OPC are required [1,2,3,4,5,6]. The reduced carbon emission geopolymers (first proposed and invented by Davidovits in 1978 [7]) have gained increasing attention around the world. Geopolymer is a tetrahedral network system composed of poly(aluminosilicate) (Si-O-Al-O) [8], which is produced by geopolymerization [9] using aluminosilicate-rich materials (e.g., fly ash [10], uncalcined coal gangue [11], slag [12], and other factory sweepings) and alkali activator. Furthermore, clayey materials and construction and demolition wastes (including ceramic waste) are also used in the geopolymerization process [13]. Given that industrial waste is used as the main raw material for geopolymer synthesis, CO_2_ emissions are greatly reduced in comparison to ordinary Portland cement [14].

How to improve the thermal insulation performance of geopolymers is currently a hot topic [15,16,17,18,19]. Previous research has found that porosity is an important factor dominating thermal conductivity [20]. Due to the increased porosity, the cross-sectional area of heat transfer in the geopolymer is reduced. Furthermore, the thermal transfer path becomes tortuous due to the presence of pores, which ultimately reduces the thermal conductivity of the geopolymer. Therefore, introducing more pores in geopolymer matrix is a potential way to improves the thermal insulation performance [21,22,23]. It has been proved that a variety of modifying agents is effective in increasing the porosity of the geopolymer. For example, hydrogen peroxide (H_2_O_2_) can be decomposed into O_2_ under alkaline conditions, and thus leaves large-diameter pores (>1 μm) during curing [24]. Moreover, phenolic resin is expanded by heating, thus facilitating the formation of a porous network structure and promoting porosity development [25]. Silica aerogel is also applied to geopolymers due to its high specific surface area and extremely low thermal conductivity [26].

Many previous studies have focused on improving the thermal insulation property of geopolymer considering using only single modified material, whereas the combined effects of various modifying agents on geopolymers have been less studied. Moreover, the thermal conductivity of the air in the pores at room temperature and constant pressure is defined as 0.026 W/m·K [27], but the micrometer and nanometer scale pores will reduce the average free path of gas and trigger the Knudsen effect [28]. This can lead to lower thermal conductivity of the pore than that at room temperature and pressure (causing deviation).

Therefore, three modifying agents were selected (phenolic resin, hydrogen peroxide, and silica aerogel) to investigate the thermal insulation performance of fly ash geopolymer. In order to optimize the number of experiments, a three-factor four-level orthogonal experiment (a method suitable for multivariate statistical analysis) was designed. Hence, a total of 16 modified fly ash geopolymer samples were prepared. The thermal conductivity of these samples was evaluated by the Transient Plane Source Method, and the porosity of these samples was determined by a Mercury Intrusion Porosimeter (MIP). In order to quantitatively describe the influence weights of the three modifying agents, variance analysis and range analysis were applied, and the best composition scheme of the three materials was determined according to range analysis. Furthermore, in order to consider the change of gas thermal conductivity caused by the Knudsen effect, an iterative calculation method was proposed to predict thermal conductivity in a more accurate way. This research explores the possibility of multiple factors for improving the thermal insulation performance of geopolymers, and analyses the influence weight of the multiple factors on the thermal insulation performance from the perspective of porosity. Moreover, an iterative calculation-based thermal conduction model is proposed in this paper which can help evaluate the thermal conductivity of geopolymer considering the micropore (or smaller)-induced Knudsen effect. In addition, the newly proposed geopolymer is significant for researchers and engineers to deploy a thermally insulating material in the construction field.

## 2. Materials and Methodology

The materials used in the experiment are fly ash, and alkali activator. The thermal insulation modification materials used are hydrophilic silica aerogel, phenolic resin, and hydrogen peroxide. The composition analysis and the loss on ignition of fly ash were provided by Yulian Power Plant in Zhengzhou City, China. Furthermore, Section 2.1 introduces a variety of physical and chemical characterization methods for geopolymer samples. Before the preparation of the sample, the microscopic composition of fly ash and three modifying materials was observed by SEM.

### 2.1. Physical and Chemical Characterizations Methodology

#### 2.1.1. FTIR

In order to investigate the chemical environments of molecular bonds in geopolymers, Fourier Transform Infrared (FTIR, Thermo Scientific NICOLE IS5 instrument from Thermo Fisher Scientific Co., Ltd. in Shanghai, China) spectroscopy was used to record the absorption of specific molecular bonds. A quantity of 50 mg powder of samples and 2 g KBr powder were mixed with a mixer. After mixing, 205 mg mixture was accurately weighed and placed in the tableting die to press the tablet. The wavenumber range ranges from 4000 to 400 cm^−1^, the spectral resolution is 2 cm^−1^, and the scanning speed is 0.2 cm^−1^/s.

#### 2.1.2. Mechanical Strength Test

The uniaxial compressive strength of geopolymer samples was tested by a TAW-2000 uniaxial pressure testing machine. A layer of lubricating oil was applied to the bottom and end of the samples (Φ50 mm × 100 mm) prior to placing them in the middle of the indenter of a servo rock mechanics testing machine to prevent the friction effect and possible shear stress during the test.

#### 2.1.3. SEM Observation

In order to visually observe the pore structure of the samples, all samples were observed by a Hitachi Regulues 8100 instrument of Hitachi Analytical Instruments (Shanghai) Co., Ltd. in Shanghai, China. Au was used as a coating material onto the samples to avoid an uneven build-up of electrons before the observation. Acceleration voltage was set to 1 kv and the observation distance was 4.7 to 4.9 mm, and then the secondary electron (SE) imaging mode was adopted. This can not only provide evidence of the geopolymerization of geopolymer, but also enable the analysis of the different pores generated by phenolic resin, silica aerogel, and hydrogen peroxide.

#### 2.1.4. Thermal Conductivity

The thermal conductivity of all samples was measured at room temperature using a DRE-2C thermal conductivity analyzer based on transient plane heat source technology. The probe was sandwiched between two pieces of the same sample (Φ50 mm × 25 mm) for testing. When the current passes through the double helix sheet, the double helix sheet will generate heat and diffuse to the sample, and the probe will record the temperature and response time. Finally, the thermal conductivity of the sample can be obtained directly by using the proposed mathematical model.

#### 2.1.5. Mercury Intrusion Porosimeter

A Mercury Intrusion Porosimeter (MIP, Poremaster 60, Anton Paar (Shanghai) Co., Ltd. in Shanghai, China.) was used to measure pores in fly ash geopolymer. The geopolymer was broken into several cubes with a volume of about 1 cm^3^, and then the mercury was pressed into the pores by pressurization. The minimum measurable aperture is about 5 nm whereas the maximum measurable aperture is about 360 μm. The maximum pressure in the experiment was about 33,000 Psia. The pore radius (r) was determined using Equation (1):

(1) $$r=\frac{-2\gamma \cos\theta }{p}$$

where *p* is the intrusion pressure, *θ* represents the contact angle, and *γ* denotes the Hg surface tension. In the experiment, the pore size distribution and porosity can be determined by using the data of input pressure, intrusive mercury volume, sample mass, and sample volume.

#### 2.1.6. Micro-Computed Tomography

Micro-computed tomography (CT) is a non-destructive and non-invasive imaging technique that provides images of the three-dimensional internal structures without damage to geopolymers. The micro-CT system used in this study was a Phoenix Nanotom S machine, the maximum voltage of X-ray was 100 kV, and the resolution of CT scan was 0.9 μm. CT scanning generates a series of grayscale images of geopolymers, and the grayscale images are denoised. Finally, the three-dimensional reconstruction of geopolymer pores can be obtained by labelling pores and throat with a ball-stick model, and the pore changes caused by the addition of modifying agents can be observed and analyzed.

#### 2.1.7. Analysis of Variance (ANOVA) and Range Analysis

The thermal conductivity, porosity and pore size distribution of the samples were analyzed by ANOVA and range analysis using SPSS 27.0.1.0 software. ANOVA analysis can be used to determine the contribution weight of the three variables (amount of phenolic resin, hydrophilic silica aerogel, hydrogen peroxide) to the variation of thermal insulation performance and pore structure. Given there is no chemical reaction between phenolic resin, silica aerogel, and hydrogen peroxide, the interaction of these factors in variance analysis is not considered. The relevant formulas are shown in Table 1.

(2) $$S_{j}=\overset{K}{\underset{k=1}{\sum }}K{\left(\bar{X_{kj}}-\bar{X}\right)}^{2},\bar{X}=\frac{1}{n}$$

(3) $$S_{T}=\overset{n}{\underset{k=1}{\sum }}K{\left(X_{k}-\bar{X}\right)}^{2}$$

(4) $$\bar{S_{A}}=S_{A}/f_{A}$$

(5) $$\bar{S_{B}}=S_{B}/f_{B}$$

(6) $$\bar{S_{C}}=S_{C}/f_{C}$$

(7) $$\bar{S_{e}}=S_{e}/f_{e}$$

(8) $$S_{e}=S_{T}-S_{A}-S_{B}-S_{C}$$

(9) $$f_{e}=n-f_{A}-f_{B}-f_{C}-1$$

(10) $$F_{A}=\bar{S_{A}}/\bar{S_{e}}$$

(11) $$F_{B}=\bar{S_{B}}/\bar{S_{e}}$$

(12) p=1−(CDF)

where *K* is variance sources, and the freedom of this experiment is 3. Moreover, 16 experimental tests were designed, so *n* equals 16. The ratio of the variance of each factor to the error variance is referred to as *F*. According to the principle of significance analysis, the calculated *F* value is compared with the critical *F* value. A larger *F* value implies a higher significance. The *p* value is calculated by the cumulative density function (CDF) of the *F* distribution; significant difference can be obtained using SPSS 27 software.

Range analysis (known as R analysis) is an intuitive analysis method. In range analysis, the optimal level and optimal combination of each factor can be given by determining the priority of the influence degree of the hypothetical factors on the experimental objectives [29]. The R reflects the sensitivity of each factor to the test results. A larger range means a greater influence of this factor on the test results, and vice versa. The corresponding equation is determined as follows:

(13) $$k_{ij}=\frac{1}{n_{j}}\overset{n_{j}}{\underset{i=1}{\sum }}K_{ij}$$

(14) $$R=\max\left\{k_{ij}\right\}-\min\left\{k_{ij}\right\}$$

*K_ij_* is the sum of the test results of the level *j* (*j* = 1, 2, 3, 4, representing 0 wt%, 3 wt%, 6 wt%, 9 wt%) in the column *i* (*i* = 1, 2, 3, representing hydrogen peroxide, aerogel, and phenolic resin, respectively), *k_ij_* is the average value of the sum of the corresponding level tests in the column of the factor; *n_j_* is the level number; max{*k_ij_*} and min {*k_ij_*} are the maximum and minimum values of the column [29]. All calculation results are retained to three decimals.

### 2.2. The Characterization of the Original Materials and Experimental Process

**Figure 1:**
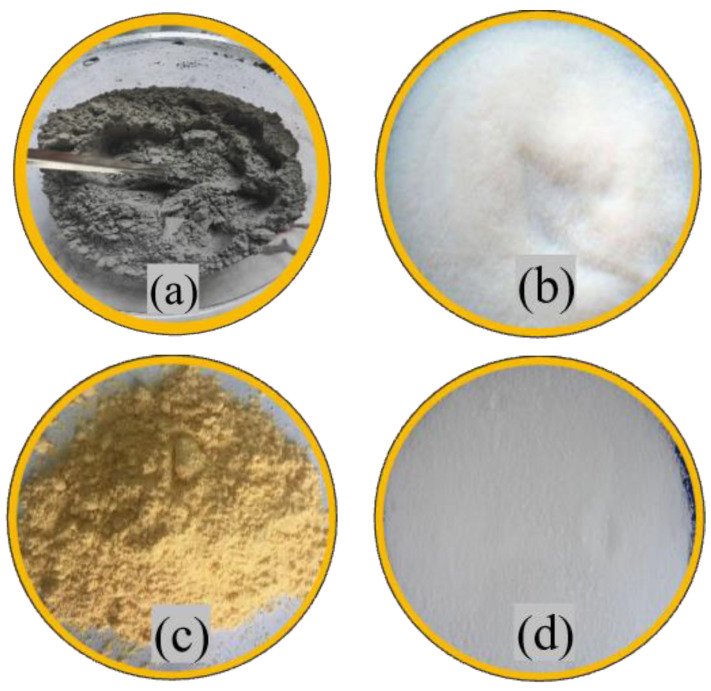
Experimental materials: (**a**) fly ash; (**b**) hydrophilic silica aerogel; (**c**) phenolic resin; (**d**) H_2_O_2_ powder.

**Figure 2:**
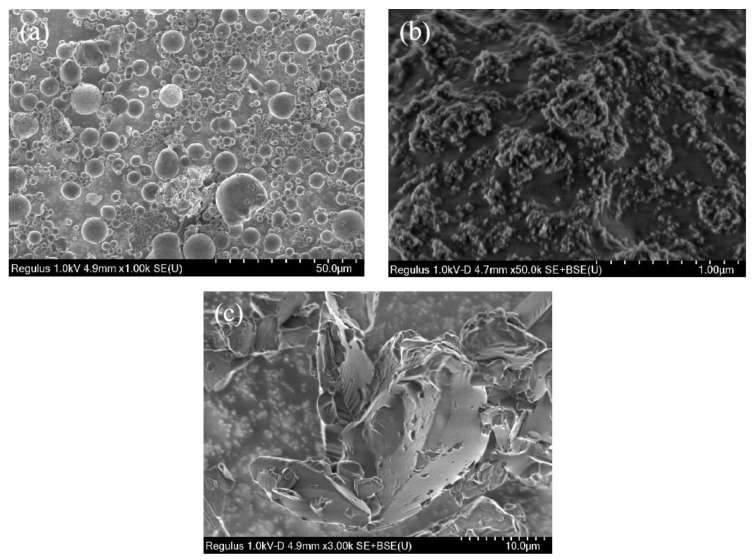
SEM images of experimental materials: (**a**) fly ash; (**b**) hydrophilic silica aerogel; (**c**) phenolic resin.

The fly ash (composition is shown in Table 2) used in the experiment is selected from Yulian Power Plant in Zhengzhou, China (Figure 1a). Fly ash consists of almost regular spheres, as identified using scanning electron microscopy (Figure 2a). The content of silica and alumina in fly ash is more than 80%, which is an excellent precursor material for geopolymer preparation (Table 2). Hydrophilic silica aerogel (Figure 1b), with a specific surface area of 500 m^2^/g, bulk density of 50 kg/m^3^, and diameter less than 20 nm) was provided by Deep Chemical Technology Co., Ltd. in Shanghai, China. Silica aerogel has very low thermal conductivity (0.02 W/m·K) and good thermal insulation performance. The silica aerogel demonstrates a ball structure via the electron microscope (Figure 2b). Solid phenolic resin powder (Figure 1c and Figure 2c, analytically pure) was provided by Henan Borun Company (Gongyi, China), with good foaming and bonding properties. H_2_O_2_ powder (Figure 1d, analytical reagent) was used as a chemical foaming agent. Sodium silicate solution (water glass, Tianjin Zhiyuan Chemical Reagent Co., Ltd. in Tianjin, China, analytical reagent) and Sodium hydroxide (Shanghai Yien Chemical Technology Co., Ltd. in Shanghai, China, solid flake, analytically pure) form an alkaline activator with SiO_2_/Na_2_O molar ratio (Ms) of 1.4; it is used for fly ash activation during geopolymerization. The silicon/aluminum molar ratio of the geopolymer is kept at 1.9 by adding alkaline activator, because this ratio is documented to be effective in producing more Si-O-Al bonds and promoting the formation of pores in the geopolymer [30].

**Figure 3:**
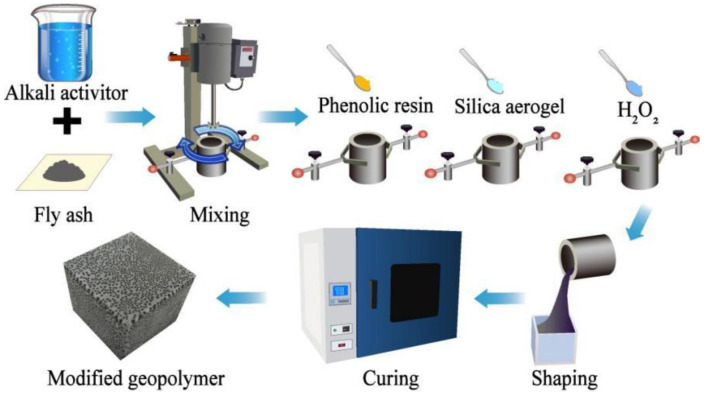
Geopolymer production process.

In order to investigate the thermal insulation effect of hydrophilic silica aerogel, phenolic resin, and H_2_O_2_, a three-factor and four-level orthogonal experimental scheme is designed (Table 3). The experimental process for synthesizing modified geopolymer is shown in Figure 3. First, the alkaline activator was added to the fly ash to make a slurry, and the slurry was then placed in a dispersion mixer for stirring (at 5 min at 400 r/min). Then, the stirring speed was increased to 700 r/min, and phenolic resin powder was slowly added. After the phenolic resin was fully mixed with the geopolymer slurry, the stirring speed was kept constant, and the hydrophilic silica aerogel was slowly added. Finally, the stirring speed was increased to 1500 r/min, and H_2_O_2_ powder was slowly poured into the slurry. After all materials were homogenously mixed, the slurry was poured into the specific mold, and all samples were sealed with a polyethylene film to prevent water loss. The samples were cured in a drying oven at 80 °C for 24 h. Finally, the samples were taken out and stored at room temperature for 7 days.

## 3. Results

### 3.1. FTIR Results of Geopolymer Samples

**Figure 4:**
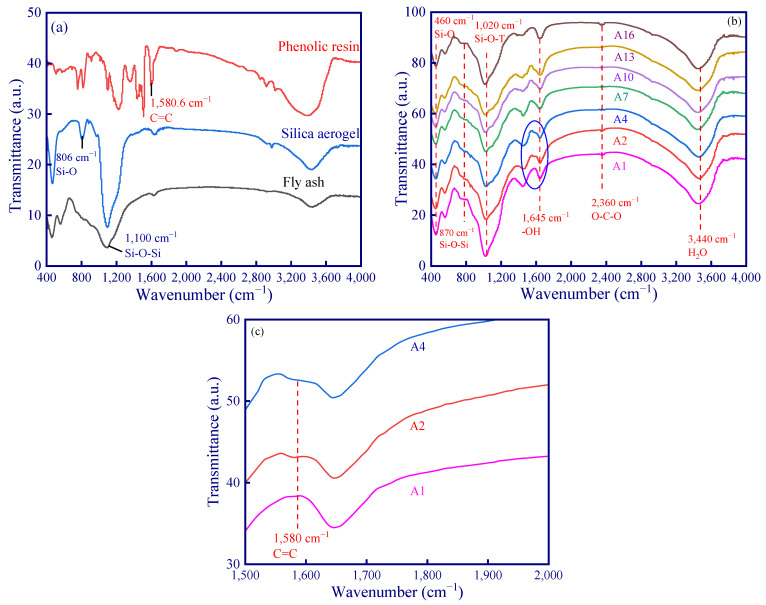
FTIR spectra: (**a**) experimental materials; (**b**) geopolymer samples; (**c**) the absorption curves of A1, A2, and A4 in the range of 1500 to 2000 cm^−1^.

The C=C stretching vibration peak of phenolic resin at 1580 cm^−1^ is clearly observed (Figure 4a). Moreover, the antisymmetric stretching vibration peaks of Si-O-Si (1100 cm^−1^) in fly ash can be identified in Figure 4a. The FTIR spectra curve of these samples is similar (Figure 4b). Firstly, a broad absorption peak of water was observed at 3440 cm^−1^, indicating the presence of unbound water in the geopolymer sample. The bending vibration peak of -OH in bound water is also obvious at 1645 cm^−1^. A small wide absorption peak was observed at 2360 cm^−1^, which was attributed by the carbonization of O-C-O [31]. The small asymmetric stretching vibration peak of CO_3_^2−^ at 1420 cm^−1^ is ascribed to the reaction of carbon dioxide with alkali metal cations migrating in geopolymer [32]. There is a wide absorption peak at 1020 cm^−1^, and it represents the asymmetric vibration of Si-O-T (T can be Si or Al) bond [32]. This is because the active substance in fly ash reacts with alkali activator, and then the Si-O-Si bond in fly ash is broken. Moreover, Si^4+^ in fly ash is substitute by Al^3+^, resulting in a silica-aluminum substance which experiences the dissolution-geopolymerization reaction [33], and the generated hydration products are the main sources of the development of geopolymer strength [32]. There is an indistinct symmetrical stretching vibration peak of Si-O-Si bond at about 870 cm^−1^, representing the residue and unreacted fly ash powder. Finally, the symmetrical variable angle vibration peak of Si-O is observed at 460 cm^−1^. It is worth noting that the stretching vibration peak of C=C was found at 1600 cm^−1^ (Figure 4c) [25], indicating that phenolic resin may not participate in the reaction in an alkaline environment (existing in the geopolymer matrix as a binder). Furthermore, the stretching vibration peak of C=C is not obvious due to a smaller amount in comparison to fly ash.

### 3.2. Compressive Strength Results of Geopolymer Samples

**Figure 5:**
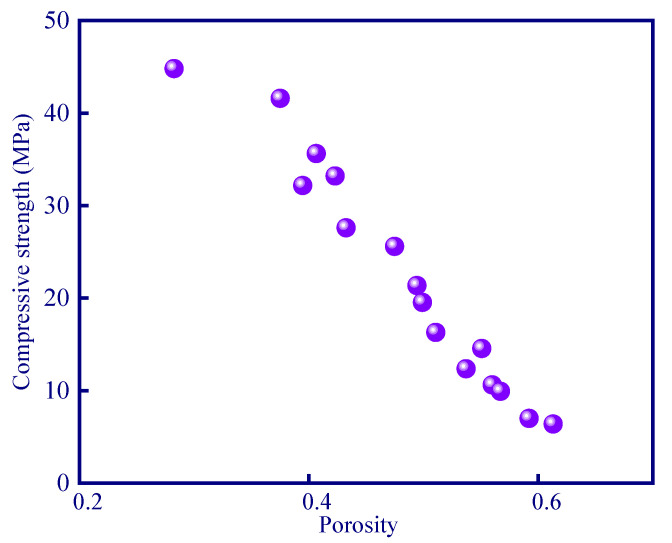
Correlation between porosity and compressive strength.

Mechanical properties are an important factor to consider when evaluating the properties of porous geopolymer materials. Compressive strength demonstrated an approximately linear relationship with respect to porosity (Figure 5). The compressive strength of the A1 sample can reach 44.8 MPa. With the addition of modifying agent (phenolic resin, hydrogen peroxide, and silica aerogel), the porosity of the sample gradually increased, and the compressive strength therefore decreased because of the newly formed pores. The results showed that the compressive strength decreased to 6.4 MPa as the porosity increased to 61.7%. It should be noted that the stable cross-linked network structure formed by phenolic resin can also benefit compressive strength development of the geopolymer [34], but the compressive strength is negatively correlated to the porosity since the addition of thermal insulation modifying agents may induce the generation of pores. Hence, the pores can trigger stress concentration under loading and lead to a decrease in compressive strength [25].

### 3.3. Analysis of Thermal Insulation Modification Effect of Samples

It is well known that the heat transfer mechanism of porous materials includes thermal radiation, thermal convection, and thermal conduction [35]. Thermal radiation occurs due to its temperature according to the radiated electromagnetic waves. Thermal convection is a heat transfer process ascribed to the relative displacement of fluid particles. Thermal conduction refers to the energy transfer of different objects at different temperatures. This research will analyze the thermal insulation modification effect of the sample considering thermal conduction. A simple and reasonable way to reduce solid thermal conduction is to replace part of the solid by gas (i.e., introducing pores into the material) [15]. This section will analyses the thermal insulation modification effect of geopolymer from the perspective of porosity and evaluate the influence weight of phenolic resin, silica aerogel, and hydrogen peroxide by variance analysis and range analysis.

#### 3.3.1. Analysis of Porosity and Thermal Conductivity of Geopolymer

**Figure 6:**
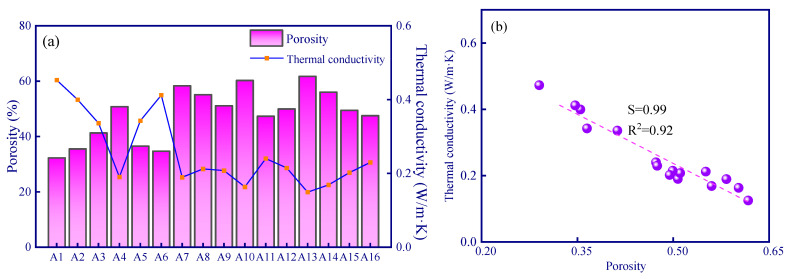
The thermal conductivity of different samples: (**a**) porosity variation-induced varying thermal conductivity; (**b**) a correlation between thermal conductivity and porosity, the slope (S) is 0.99.

**Figure 7:**
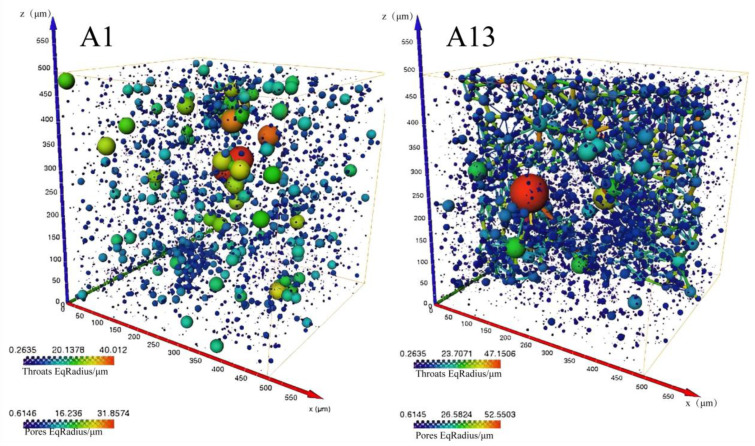
Three-dimensional tomography of some samples.

Since the thermal conductivity of the gas in the pores is much lower than that of the geopolymer matrix, a larger porosity-induced lower thermal conductivity of the sample can be expected (Figure 6a,b). The porosity of the A13 sample was the lowest, which resulted in a thermal conductivity of 0.12 W/m·K. In addition, the sample with the highest thermal conductivity was the A1 sample (0.48 W/m·K), and the porosity of the A1 sample (control experiment, fly ash geopolymer without any modified material) was 28.2% (Figure 6a). The thermal conductivity is negatively correlated with porosity (a roughly linear relationship). During the solidification of the geopolymer at constant temperature, a certain number of micro-pores and nano-pores are generated via the evaporation of water inside the material, and the porosity is about 25~30% [36]. The porosity of the modified geopolymer has been increased to a large extent due to the addition of modifying agents in comparison to the A1 sample. Among the samples, the porosity of the A13 sample is the highest which can reach to 61.7%. The micro-computed tomography image demonstrated the internal pore structure of modified geopolymer (A13 sample) in comparison to the control experiment (A1 sample), and considerably developed pores can be observed (Figure 7).

**Figure 8:**
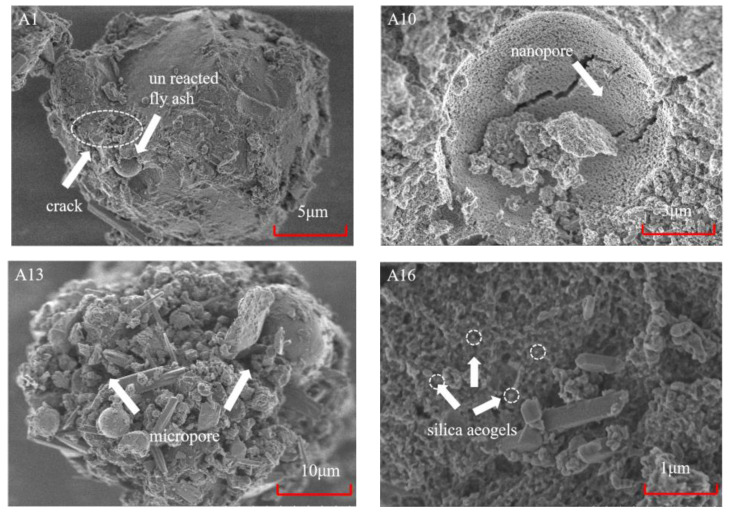
SEM images of geopolymer samples (A1, A10, A13, and A16 samples, respectively).

The geopolymer matrix (A1 sample) is relatively dense due to its low porosity, and unreacted fly ash particles can be seen. A small amount of cracks are distributed onto the geopolymer surface, which can be clearly seen in the A10 sample (Figure 8). In the A10 and A13 samples, modifying agents of hydrogen peroxide, phenolic resin, and silica aerogels were added. It can be found that the A10 sample produces many nanopores compared to the A1 sample since hydrogen peroxide reacts with alkali activator to produce O_2_ and the silica aerogel is embedded in the geopolymer (silica aerogel is characterized by a hollow microsphere structure, which can be treated as nanopores) (Figure 8). The micropores significantly increased and developed in the A13 sample. It can be highly speculated that the accumulation of oxygen (generated after the addition of hydrogen peroxide) facilitates the formation of larger pores [37]. Additionally, phenolic resin can promote the formation of a cross-linked porous mesh structure after elevated temperature (above 80 °C) curing [38]. It should be noted that open pores also affect the thermal conductivity through water absorption [39]. The thermal conductivity of penetrated water in geopolymers through open pores is higher than that of gas in pores, thus resulting in an increase in overall thermal conductivity. Therefore, the water-proof property of geopolymers should be further improved in future work.

#### 3.3.2. Analysis of Variance and Range Analysis for Porosity and Thermal Conductivity

The influence weight of the three factors (phenolic resin, hydrogen peroxide, and silica aerogel) on the thermal insulation performance cannot be simply analyzed solely considering porosity, and mathematical statistics are required. In this section, the dependent variable is porosity, and the independent variables are the additions of three modifying agents. Range analysis and variance analysis are used to determine the contribution weight of the three independent variables to the porosity growth, and the best theoretical mixing ratio for a low thermally insulated geopolymer is then derived. The results of the two analysis methods are introduced as follows.

**Figure 9:**
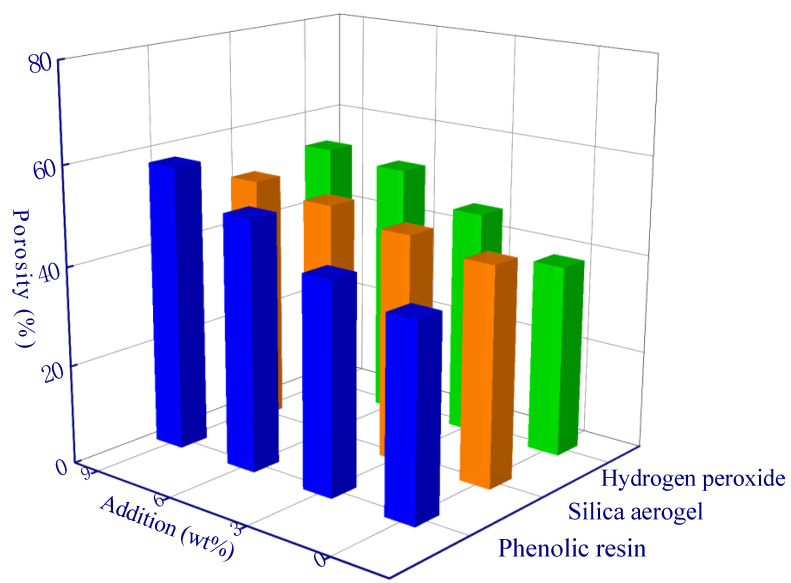
Range analysis of porosity.

**Figure 10:**
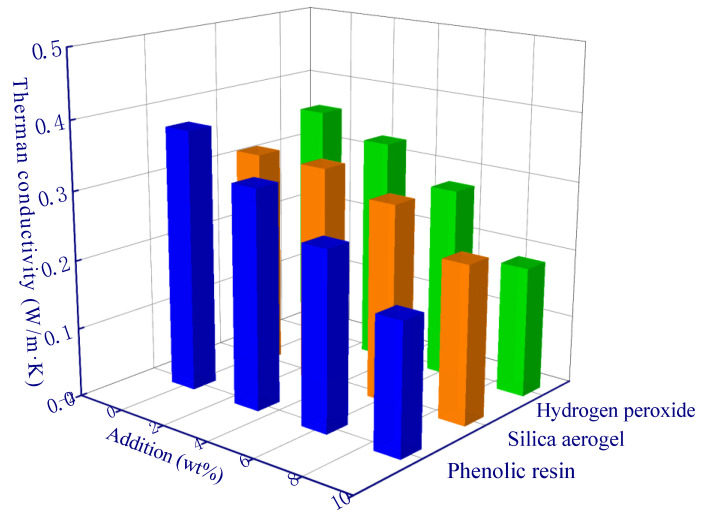
Range analysis of thermal conductivity.

Table 4 and Table 5 are the three-factor range analysis tables of porosity and thermal conductivity. The role of *R* value and *K* and *K_avg_* values in the table is used to determine the priority of the influence degree of hypothetical factors (phenolic resin, silica aerogel, and hydrogen peroxide) on experimental objectives (porosity and thermal conductivity), and the optimal level and optimal combination of each factor can be deduced, respectively [29]. The *K_avg_* values of the three factors in the Table 4 and Table 5 generally follow a linear trend, and all peaks occur when the addition amount is the largest. For example, the *K_avg_* value of H_2_O_2_ increased from 38.9% to 53.6% (Figure 9), and the thermal conductivity decreased from 0.36 W/m·K to 0.19 W/m·K (Figure 10), which reflects the effective modification effect of H_2_O_2_. The optimal levels are derived from the range analysis results in Table 4 and Table 5. The newly prepared sample is characterized with a porosity of up to 71.9% and a thermal conductivity as low as 0.08 W/m·K using the optimal theoretical mixing ratio. Hence, the rationality of range analysis is verified by the experimental results.

The specific calculation of variance analysis was completed by SPSS 27 software. The main processing results include sum of squares, degree of freedom, mean square, *F* value, and *p* value. A smaller *p* value denotes a greater significance (Table 6 and Table 7). The ability to produce porosity increases in the order: phenolic resin < H_2_O_2_ < silica aerogel (Table 6). The ability to lower thermal conductivity increases in the order: phenolic resin < H_2_O_2_ < silica aerogel (Table 7). As an excellent thermal insulation material, the significance of silica aerogel is 0.083 and it does not show obvious significance compared with the other two modifying agents in the experiments (Table 7). This is probably due to the chemical reaction between aerogel and alkaline activator, thus leading to the resultant silicate. As a result, fewer aerogel particles can occur in the geopolymer matrix, which decreases the thermal insulation effect of silica aerogel to a minor extent.

### 3.4. Analysis of Pore Size Distribution of Samples

#### 3.4.1. Analysis of Fractal Geometry of Samples

In addition to the relationship between porosity and thermal conductivity, some scholars have found that pore size distribution has an impact on thermal conductivity [40]. Moreover, fractal geometry has been proved effective in determining the pore size distribution of porous materials [41,42,43,44,45]. In order to explore the influence of the three factors on pore size distribution, the research uses a thermodynamic fractal model to derive fractal dimension (for quantifying the change of pore size). Then, the variance analysis of fractal dimension is used to evaluate the significance of the three factors to the pore fractal. The fractal dimension reflects the heterogeneity of pore structure, and the corresponding result usually locates between 2–3. A larger fractal dimension represents a more complex pore structure [46]. According to the MIP experiments, the volume of mercury gradually increases as the injection pressure increases. The relationship between mercury increment and pore surface energy can be described by the following Equation (15):

(15) $$dW=-PdV=-r_{L}\cos\theta \;dS$$

where *dW* stands for the surface energy, *θ* denotes the contact angle between mercury and pore surface (about 140° in the experiments), *r_L_* represents the surface tension between mercury and surface, and *S* is the surface.

Furthermore, the increments of mercury crushed into the pores (*Q_n_*) with respect to the surface energy (*W_n_*) can be determined as Equation (16):

(16) $$\ln(W_{n})=\ln(Q_{n})+C$$

Moreover, Zhang et al. [47] modified Equation (16) to avoid using trial and error:

(17) $$\ln(W_{n}/r_{n}{}^{2})=D\ln(V_{n}{}^{1/3}/r_{n})+C$$

where *V_n_* is the pore volume; *r_n_* is the pore radius; *D* is the slope of Equation (17), which represents the surface fractal dimension.

**Figure 11:**
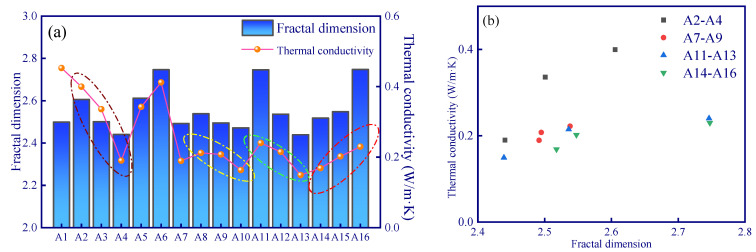
(**a**) Thermal conductivity and thermodynamic fractal dimension of samples; (**b**) relation between thermal conductivity and thermodynamic fractal dimension of samples.

**Figure 12:**
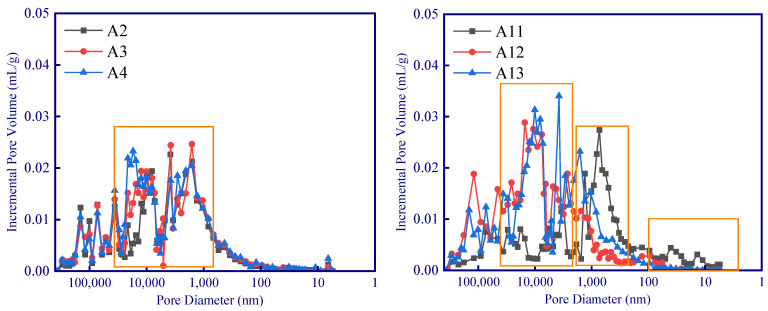
Pore size distribution of geopolymer samples.

For all these samples, no obvious correlation between fractal dimension and thermal conductivity can be observed since the pore size distribution between various samples is quite different. However, the fractal dimension and thermal conductivity of samples with similar pore size distribution (as the colored circle shown in Figure 11) demonstrated a linear relationship (Figure 11b). In addition, the slopes of these linear relationships are not the same (Figure 11b). A large difference in pore distribution between samples can result in a significant difference in the range of pore complexity or homogenization (as shown in Figure 12, the A2–A3 samples have obvious pore changes in the range of 1000–10,000 nm, while A11–A13 have obvious pore changes in the range of 10–10,000 nm). Given that different sizes of pores have different effects on the thermal insulation performance (Knudsen effect), different correlations between the fractal dimension and the thermal conductivity of the samples in different experimental groups can be observed (Figure 11a).

The variance analysis of fractal dimension can help to analyze the influence of the three factors on pore size distribution and then to assess the influence on macro thermal insulation performance. The significant difference (*p*) increases in the order: (H_2_O_2_) < (Phenolic resin) < (silica aerogel). Among the three factors in Table 8, only hydrogen peroxide has significant difference (*p* < 0.05), indicating that hydrogen peroxide will simply decrease the complexity of pores in geopolymer. Moreover, phenolic resin and aerogel have no significant effect on fractal dimension. Combined with the variance analysis results of porosity in Table 6, both of phenolic resin and silica aerogel have an effect on the ultimate porosity. It is speculated that the influence of the two factors may have different impacts in different pore size ranges. In a specific pore size range, they can promote pore growth, whereas these two factors inhibit pore formation in another range. Hence, the competition of promoting and inhibiting effects results in less variation in the overall fractal dimension by using phenolic resin and silica aerogel (analyses in the following section).

**Figure 13:**
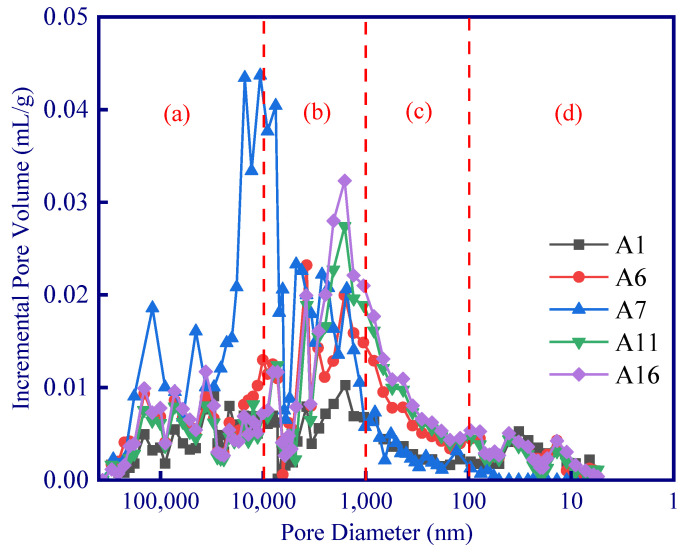
Classification of pore size distribution range of selected geopolymers: (a) >10 μm; (b) 1–10 μm; (c) 100 nm–1 μm; (d) <100 nm.

In order to analyze the influence of hydrogen peroxide, phenolic resin, and silica aerogel on different pore size, it is necessary to analyze the pore distribution in detail. According to the variation of Incremental Pore Volume, the pore distribution can be divided into four ranges: (a) >10 μm, (b) 1–10 μm, (c) 100 nm–1 μm, and (d) <100 nm (Figure 13). Therefore, variance analysis and range analysis are carried out to analyze the impact of the three modifying agents on porosity in the four pore size ranges.

#### 3.4.2. Variance Analysis and Range Analysis of Each Pore Size Range of Samples

**Figure 14:**
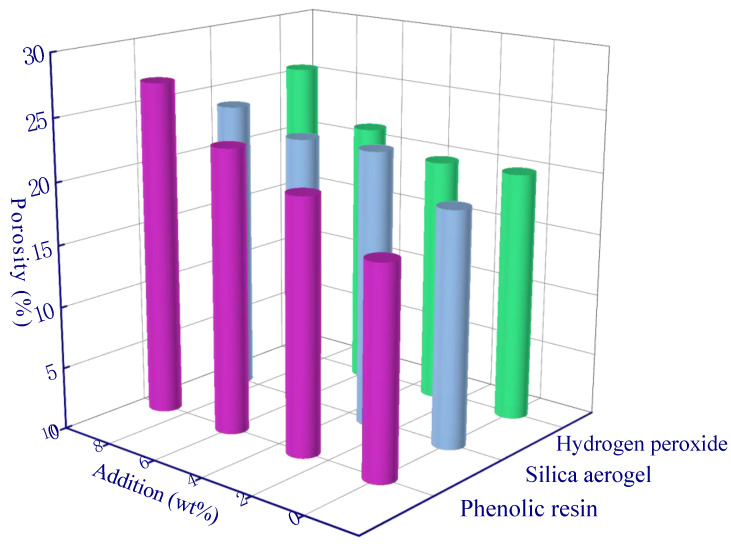
Range analysis of pore (>10 μm).

**Figure 15:**
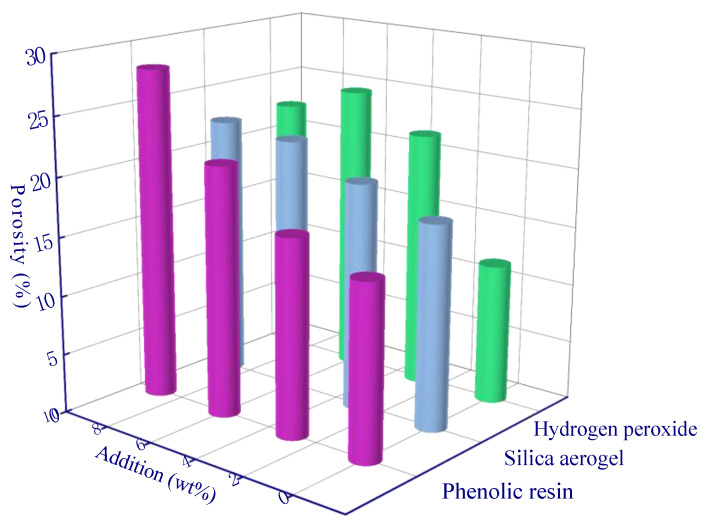
Range analysis of pore (1–10 μm).

**Figure 16:**
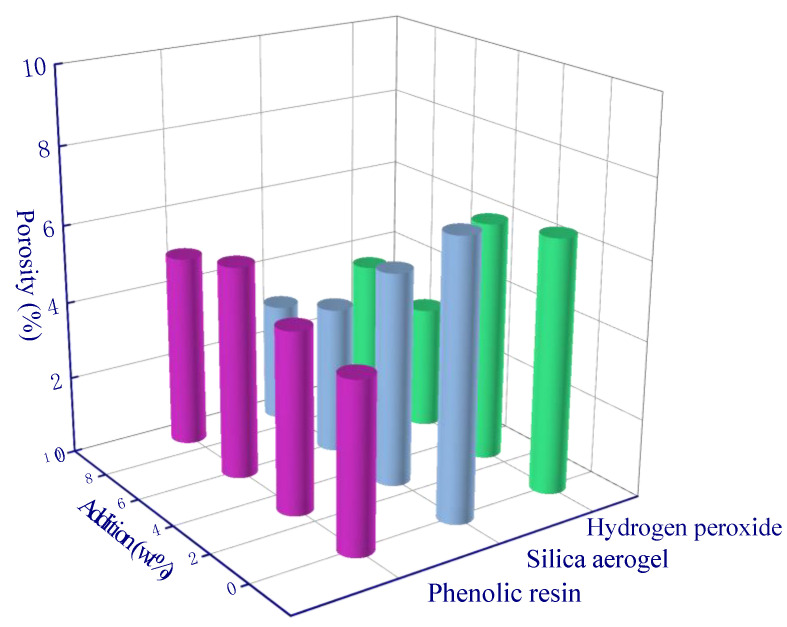
Range analysis of pore (100 nm–1 μm).

**Figure 17:**
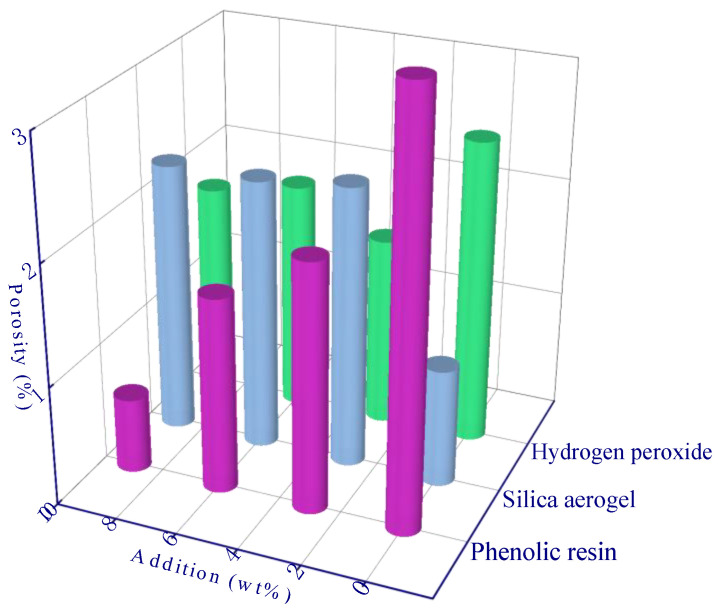
Range analysis of pore (<100 nm).

The ANOVA in each aperture range is presented in Table 9, Table 10, Table 11 and Table 12, and the range analysis in each aperture range is shown in Figure 14, Figure 15, Figure 16 and Figure 17. In the pore size range of >10 μm, there was a significant difference between phenolic resin and hydrogen peroxide (the phenolic resin effect outweighed the hydrogen peroxide effect) (Figure 14). The modification of phenolic resin increased the porosity from 16.9% to 27.2% (61% increase), and hydrogen peroxide increased from 20.1% to 25.7% (28% increase) (Figure 14). This indicates that the two modifying agents have considerable effects in improving porosity in the pore size range of >10 μm. In addition, it is also noted that the addition of silica aerogels also leads to a small increase in porosity. Aerogel is characterized by a pore size of 20 nm, and it will not theoretically impact the pores of >10 μm, whereas the aerogel (SiO_2_) reacts with the OH^−^ in the alkali activator and forms water, which will produce more macropores during the isothermal curing process [48].

For the pore size range of 1–10 μm, phenolic resin and hydrogen peroxide also showed significant differences, indicating that more pores were mainly produced by these two modifying agents in this pore range. The porosity using phenolic resin increased from 14.8% to 28.1% (Figure 15), and the increase rate (90%) was greater than that at >10 μm (61%), indicating that phenolic resin dominated the formation and growth of pores in the range 1–10 μm. If the amount of hydrogen peroxide added reached 6 wt%, a maximum porosity is observed at 24.6%, and then it decreased to 22.5% as the hydrogen peroxide amount increased to 9 wt%. This phenomenon shows that a higher concentration of hydrogen peroxide can produce a large amount of O_2_ in a short time and cause an accumulation in the geopolymer slurry, thus leaving larger diameter pores (e.g., >10 μm pores as shown in Figure 14) and promoting smaller pore growth (e.g., 1–10 μm pores as shown in Figure 15) to a lesser extent.

In the pore range of 100 nm–1 μm, hydrogen peroxide, phenolic resin, and silica aerogel may not show an obvious significance (Table 11). Moreover, the modification effect of the three factors on porosity is insignificant according to the range analysis (Figure 16). The three modifying agents have negligible effect on porosity in the range of 100 nm–1 μm, which enable a new insight for future research. A material which can also significantly increase porosity in the range of 100 nm–1 μm is more promising, and the thermal insulation performance of the sample can be further improved.

For the pore size of <100 nm, both phenolic resin and silica aerogel have a significant impact on porosity. The silica aerogel can improve the porosity in the pore range of <100 nm. When the addition amount is 3 wt%, the porosity increases from 0.9% to 2.35%. However, the porosity slightly varies as the added silica aerogel amount continuously increases. This is because a large amount of aerogel can react with alkali activators, thus destroying the microporous structure of aerogel and resulting in less aerogel embedded in the geopolymer.

### 3.5. Optimization and Modification of Thermal Conductivity Model of P orous Geopolymer

Based on thermal conductivity and porosity measured by MIP experiments, multiple analytical models can be used to predict the effective thermal conductivity of porous solids by considering pore volume fraction. In order to provide a more accurate thermal conductivity model, this section will propose an iterative calculation method for thermal conductivity prediction.

As a new inorganic cementitious material rich in Si and Al, the composition of geopolymer includes a mixed matrix of aluminosilicate material with an amorphous 3D structure [49], as well as nano-to-micron size pores and cracks. Due to the large range of pore size distribution (according to Section 2.1.5) and the complex solid composition, it is challenging to propose an analytical model to determine the effective thermal conductivity of porous geopolymer materials. In order to simplify the calculation, the geopolymer is firstly regarded as a bicomponent model composed of solid and gas. This can facilitate the calculation of the thermal conduction. In this bicomponent model, the phenolic resin is considered as a part of the solid material, and this research only considers the pores generated by hydrogen peroxide (hydrogen peroxide was fully reacted to produce O_2_). Since the structure of the hollow silica aerogel microspheres is similar to the pore, it can then be treated as the gas phase. Secondly, an iterative calculation method considering the Knudsen effect is proposed to calculate the thermal conductivity more accurately.

In this research, the thermal conductivity of fly ash based geopolymer matrix (without chemical modification) in this experiment is 0.55 W/m·K [27]. Moreover, the thermal conductivity of phenolic resin is 0.2 W/m·K [50], and the thermal conductivity of gas in pores is 0.026 W/m·K [27]. Previous studies [20] have shown that the ratio of the thermal conductivity of the solid component to the gas component is much larger than 1, higher porosity by increasing the volume of the gas component can significantly reduce the overall thermal conductivity. Hence, the porosity will have a significant effect on the thermal conductivity.

According to Skochdopole [51] and Stefan–Boltzman’s law, thermal convection and radiation can be ignored if the aperture is less than 1 mm. Therefore, a variety of thermal conduction models were selected to calculate the thermal conductivity. As the porous geopolymers are usually considered as a continuous solid phase with a uniformly dispersed cavity for filling fluid (air or other gas), hence, a Maxwell–Euchen model is proposed considering randomly distributed pores with different diameters (Equation (18)) [52]:

(18) $$\lambda =\lambda_{p}\frac{2\lambda_{p}+\lambda_{f}-2V_{f}\left(\lambda_{p}-\lambda_{f}\right)}{2\lambda_{p}+\lambda_{f}+V_{f}\left(\lambda_{p}-\lambda_{f}\right)}$$

where *λ* is the thermal conductivity of geopolymer, *λ_p_* represents the thermal conductivity of solid components, *λ_f_* denotes the thermal conductivity of gas composition, *V_f_* stands for porosity.

Effective Medium Theory (EMT) is a thermal conduction model based on heterogeneous two-component materials [27], as shown in Equation (19):

(19) $$\lambda =\frac{ [3V_{f}-1]\lambda_{f}+ [2-3V_{f}]\lambda_{p}+\sqrt{{\left\{ [3V_{f}+1]\lambda_{f}+ [2-3V_{f}]\lambda_{p}\right\}}^{2}+8\lambda_{f}\lambda_{p}}}{4}$$

Additionally, taking into account the different shapes of pores, Russell [53] deduced the thermal conductivity model of pores by using series formula and parallel formula (Equation (20)):

(20) $$\lambda =\lambda p\frac{ [{Vf}^{\frac{2}{3}}+\frac{\lambda p}{\lambda f}\left(1-{Vf}^{\frac{2}{3}}\right)]}{ [{Vf}^{\frac{2}{3}}-Vf+\frac{\lambda p}{\lambda f}\left(1-{Vf}^{\frac{2}{3}}+Vf\right)]}$$

Cheng [54] constructed a thermal conduction model for two-phase mixture which is suitable for porous materials (Equation (21)):

(21) $$\frac{1}{\lambda }=\frac{\sqrt{V_{f}}}{\lambda_{p}+(\lambda_{p}+\lambda_{f})\sqrt{V_{f}}}+\frac{1-\sqrt{V_{f}}}{\lambda_{p}}$$

In addition, the parallel model (Equation (22)) and the series model (Equation (23)) were constructed based on multi-shaped filler structure [55]:

(22) $$\lambda =\left(1-V_{f}\right)\lambda_{p}+V_{f}\lambda_{f}$$

(23) $$\lambda =\frac{1}{\left(1-V_{f}\right)/\lambda_{p}+V_{f}/\lambda_{f}}$$

**Figure 18:**
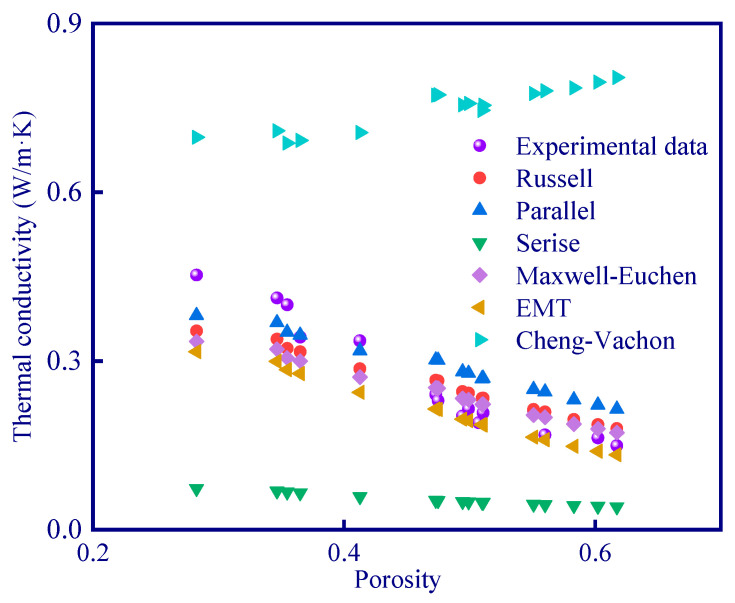
Fitting results of various thermal conduction models.

**Figure 19:**
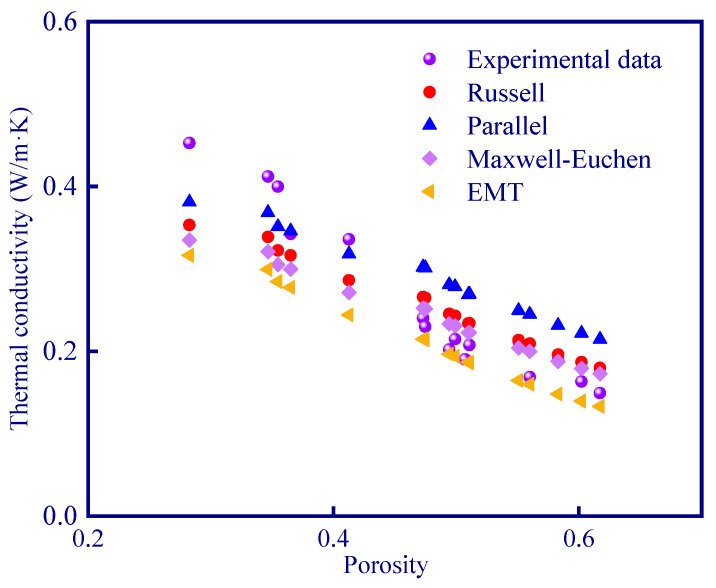
Fitting results of four thermal conduction models.

Among the various thermal conductivity models, it can be seen that only the series model and Cheng–Vachon model have a large deviation from the experimental data (Figure 18). This is because the Cheng–Vachon model is suitable for composites with higher thermal conductivity of dispersed phase (discontinuous material in the system, the dispersed phase in geopolymer is pore) than that of continuous phase (continuous material in the system, continuous phase is geopolymer matrix in this study), while the series model is suitable for materials with extremely low thermal conductivity. In order to quantitatively evaluate the fitting effect of the thermal conduction models (Figure 19), a parameter ‘average deviation’ (AD) is defined in this study (Equation (24)):

(24) $$AD=\frac{\sum_{i=1}^{16}\left|N_{i}-I_{i}\right|}{n}$$

where *I_i_* is the experimental result for the *i*th orthogonal experiments, *N_i_* is corresponding calculated result.

The AD is the average of absolute differences between the calculated results and the experimental results. A smaller AD represents a smaller average deviation, indicating that the calculated result is close to the experimental data (a better fitting effect can then be expected, and vice versa).

Table 13 shows the average deviation of each thermal conduction model. The average deviation of the Cheng–Vachon model and the series model is high, indicating that these two models are not suitable for describing the thermal conductivity of porous geopolymer. The other four models fit well with the experimental data, and the best one is the Maxwell–Euchen model which has an average deviation of 0.038 W/m·K.

It is worth noting that although the calculation results of the Maxwell–Euchen model are very close to the experimental results, there are still deviations. This is because the reduction in pore size (the presence of micron- or even nanometer-scale pores) will trigger the Knudsen effect (Equations (25) and (26)) [56].

(25) $$\lambda gas=\frac{\lambda gas,0}{1+2\beta Kn}=\frac{\lambda gas,0}{1+\frac{\sqrt{2}}{\pi d^{2}p\delta }\beta k_{B}T}$$

(26) $$Kn=\frac{\sigma_{mean}}{\delta }=\frac{k_{B}T}{\sqrt{2}\pi d^{2}p\delta }$$

where *λ_gas__,0_* stands for the gas thermal conductivity at standard temperature and pressure, *λ_gas_* is the thermal conductivity of the gas in the pore, *k_B_* stands for the Boltzmann constant, *β* is a parameter that takes into account the energy transfer between gas molecules and the limiting solid structure (ranges from 1.5 to 2.0), *σ_mean_* is the average free path of the gas molecule, *δ* is the pore size, *T* is the kelvin temperature, and *P* is the gas pressure.

The average free path of gas molecules in the pore will be reduced due to the reduction in pore size if the pore size is reduced to micrometer or even nanometer scale, thus resulting in the actual thermal conductivity being much lower than the thermal conductivity of air at room temperature. The thermal conductivity in pores is 0.021–0.025 W/m·K with a pore size of 1–10 μm [56], 0.008–0.021 W/m·K is expected as the pore size decreases to the range of 100 nm–1 μm, and 0.002–0.008 W/m·K is ultimately reached if the pore size is smaller than 100 nm. Therefore, the thermal conductivity of the pores cannot be simply regarded as the air thermal conductivity in order to obtain more accurate calculation results. Hence, this paper proposes an iterative calculation method which can consider the Knudsen effect in the thermal conductivity prediction.

Iterative calculation is a typical method in numerical calculation, which is applied to the solution of equations and the solution of matrix eigenvalues [57]. According to Kiil’s research (2015) [58], an iterative calculation method was used to solve the thermal conductivity of a three-component material (pure epoxy, pure aerogel, and aerogel intruded by epoxy). Firstly, they used the Hamilton and Crosser [59] model to calculate the thermal conductivity of a two-component material consisting of pure aerogels and aerogels invaded by epoxy resin. Then, the same thermal conductivity model was used to simulate the thermal conductivity of a two-component material (pure aerogels and aerogels invaded by epoxy resin) and pure epoxy resin.

Therefore, the Maxwell–Euchen model was chosen as the modified model (because of its lowest average deviation). In order to take into account the Knudsen effect, we divide all pores with the same pore size into one category. Hence, all pores are divided into *n* categories (*n* refers to the number of different diameters of pores in the MIP). The porous geopolymer can be divided into solid components (including geopolymer matrix and phenolic resin, the overall solid thermal conductivity can be directly calculated) and *n* types of pores with the same pore size (each type of pore has porosity *V_fn_*). During the calculation, the solid composition is firstly combined with the first type of pores (*n* = 1) with the same pore size to form a two-component structure, and then the Maxwell–Euchen model is used to calculate its thermal conductivity *λ*_1_. Then, *λ*_1_ is regarded as a new solid phase, and the two-component structure is formed again with the second type of pores with the same pore size (*n* = 2). The Maxwell–Euchen model is used again to calculate its thermal conductivity *λ*_2_, and this iterative calculation is repeated until *λ_n_* is calculated, and *λ_n_* is the ultimate thermal conductivity considering the Knudsen effect. Hence, the ultimate thermal conductivity can be calculated using Equations (27) and (28):

(27) $$\lambda_{n}=\lambda_{n-1}\frac{2\lambda_{n-1}+\lambda_{ngas}-2V_{fn}\left(\lambda_{n-1}-\lambda_{ngas}\right)}{2\lambda_{n-1}+\lambda_{ngas}+V_{fn}\left(\lambda_{n-1}-\lambda_{ngas}\right)}$$

(28) $$V_{fn}=\frac{V_{ngas}}{Vs+\sum_{i=1}^{n}V_{i(gas)}}$$

**Figure 20:**
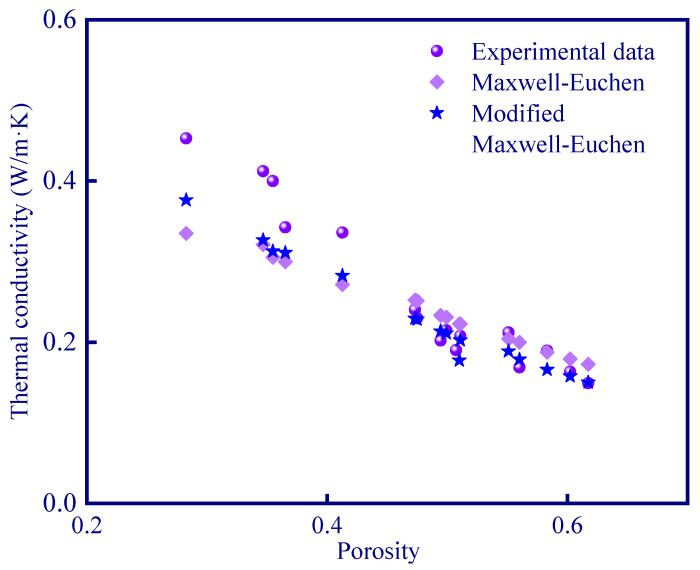
The modified Maxwell–Euchen model considering the Knudsen effect.

The thermal conductivity considering iterative calculation agrees well with the experimental results. According to Figure 20, the average deviation of Maxwell–Euchen model is reduced from 0.038 W/m·K to 0.027 W/m·K, which is more consistent with the experimental data. It is also proved that the calculation of the thermal conduction model after considering the Knudsen effect enables a more accurate prediction. Moreover, the iterative calculation method shows a more accurate calculation for high porosity samples (>45%). This is because high porosity means a more complex pore distribution, which makes the Knudsen effect less negligible.

## 4. Conclusions

In this research, three thermal insulation modifying agents (phenolic resin, silica aerogel, and hydrogen peroxide) were selected to carry out a three-factor and four-level orthogonal experiment, and the improved thermal insulation performance of geopolymer by increasing porosity is analyzed in detail. In addition, the influence weights of the three modifying agents on porosity, thermal conductivity, and fractal dimension were investigated by variance analysis and range analysis. Moreover, an iterative method considering the Knudsen effect is innovatively proposed to predict thermal conductivity of the geopolymer. Based on the above experimental results and analyses, the main conclusions can be drawn as follows:

All three thermal insulation modifying agents have a significant effect on the thermal conductivity of geopolymer. The thermal conductivity of the geopolymer decreased from 0.48 W/m·K to 0.12 W/m·K, and the corresponding porosity increased from 28.2% to 61.7%. According to the range analysis of thermal conductivity and porosity, an optimal composition scheme of the three modifying agents was applied to prepare the geopolymer, and the thermal conductivity reduced to 0.08 W/m·K with porosity of 71.9%. Analysis of variance showed that phenolic resin, hydrogen peroxide, and silica aerogel had significant effects on porosity increase (*p* < 0.05), and the significance decreases in the order: phenolic resin > hydrogen peroxide > silica aerogel.

The fractal dimension and thermal conductivity have a linear correlation (pores with similar size distribution), and only hydrogen peroxide has a significant difference in the fractal dimension of geopolymer. The effect of the three thermal insulation-modifying agents on porosity is obtained: (a) phenolic resin can increase the pores with a diameter of >1 μm and reduce the pores with a diameter of <100 nm; (b) hydrogen peroxide can increase pores with a diameter of >1 μm; (c) silica aerogel can increase pores with a diameter of >1 μm (formed by evaporation of water generated by the reaction of silica aerogel with alkali activator during curing), and increase pores of <100 nm.

A variety of thermal conductivity models were selected to predict the experimental results. It was found that the experimental data can be roughly predicted using the Maxwell–Euchen model. A better prediction of thermal conductivity of geopolymer can be achieved considering the Knudsen effect, and an iterative calculation method is proposed in this paper, and a more accurate and newly modified Maxwell–Euchen model is constructed by iterative repeated calculation. The results demonstrate that the average deviation between the modified Maxwell–Euchen model and the experimental data is reduced from 0.038 W/m·K to 0.027 W/m·K, which validates the accuracy of the iterative calculation.

## Figures and Tables

**Table 1:** Variance analysis table.

Target Parameter	Source of Variance	Quadratic Sum	Freedom	Variance	*F*	Significant Difference (*p*)
porosity and other parameters	A	*S_A_*	*f_A_ =* r *−* 1	$\bar{S_{A}}=S_{A}/f_{A}$	$F_{A}=\bar{S_{A}}/\bar{S_{e}}$	Equation (12)
	B	*S_B_*	*f_B_ =* r *−* 1	$\bar{S_{B}}=S_{B}^{}/f_{B}$	$F_{B}=\bar{S_{B}}/\bar{S_{e}}$	
	C	*S_C_*	*f_C_ =* r *−* 1	$\bar{S_{C}}=S_{C}/f_{C}$	$F_{C}=\bar{S_{C}}/\bar{S_{e}}$	
	error	*S_e_*	*f_e_*	$\bar{S_{e}}=S_{e}/f_{e}$		
	summation	*S_T_*	*f_T_ = n −* 1			

**Table 2:** Chemical composition and loss on ignition of fly ash (%).

Oxides	SiO_2_	Al_2_O_3_	Fe_2_O_3_	CaO	K_2_O	TiO_2_	MgO
amount (%)	53.968	31.148	4.160	4.012	2.035	1.133	1.011

Note: loss on ignition is 2.2%.

**Table 3:** Experimental group setting (wt%).

ID	H_2_O_2_	Silica Aerogel	Phenolic Resin
A1	0	0	0
A2	0	3	3
A3	0	6	6
A4	0	9	9
A5	3	0	3
A6	3	3	0
A7	3	6	9
A8	3	9	6
A9	6	0	6
A10	6	3	9
A11	6	6	0
A12	6	9	3
A13	9	0	9
A14	9	3	6
A15	9	6	3
A16	9	9	0

**Table 4:** Range analysis table of porosity.

Parameter	Level (wt%)	H_2_O_2_ (%)	Silica Aerogel (%)	Phenolic Resin (%)
*K*	0	155.7	177.5	157.7
	3	184.6	186.3	171.3
	6	208.5	196.3	203.4
	9	214.6	203.2	230.9
*K_avg_*	0	38.9	44.3	39.4
	3	46.1	46.5	42.8
	6	52.1	49.1	50.8
	9	53.6	50.8	57.7
Optimal levels		4	4	4
*R*		14.7	6.4	18.3
Number of levels		4	4	4
*r*		4	4	4

**Table 5:** Range analysis table of thermal conductivity.

Parameter	Level (wt%)	H_2_O_2_ (%)	Silica Aerogel (%)	Phenolic Resin (%)
*K*	0	1.45	1.28	1.52
	3	1.3	1.26	1.29
	6	1.12	1.15	1.04
	9	0.74	0.92	0.76
*K*_*avg*_	0	0.36	0.32	0.38
	3	0.33	0.32	0.32
	6	0.28	0.29	0.26
	9	0.19	0.23	0.19
optimal levels		4	4	4
*R*		0.18	0.09	0.19
number of levels		4	4	4
*r*		4	4	4

**Table 6:** Variance analysis of porosity.

Target Parameter	Source of Variance	Quadratic Sum	Freedom	Variance	*F*	Significant Difference (*p*)
	H_2_O_2_	375.412	3	125.137	18.566	0.002
	silica aerogel	119.188	3	39.729	5.894	0.032
porosity	phenolic resin	981.958	3	327.319	48.562	<0.001
	error	40.441	6	6.74		
	summation	40069.831	16			

**Table 7:** Variance analysis table of thermal conductivity.

Target Parameter	Source of Variance	Quadratic Sum	Freedom	Variance	*F*	Significant Difference (*p*)
	H_2_O_2_	0.069	3	0.023	16.628	0.003
	silica aerogel	0.015	3	0.005	3.653	0.083
thermal conductivity	phenolic resin	0.075	3	0.025	18.137	0.002
	error	0.008	6	0.001		
	summation	1.511	16			

**Table 8:** Variance analysis of fractal dimension.

Target Parameter	Source of Variance	Quadratic Sum	Freedom	Variance	*F*	Significant Difference (*p*)
	H_2_O_2_	0.111	3	0.037	8.545	0.014
	silica aerogel	0.013	3	0.004	0.977	0.464
fractal dimension	phenolic resin	0.015	3	0.005	1.147	0.404
	error	40.441	6	6.74		
	summation	40069.831	16			

**Table 9:** Variance analysis of pore (>10 μm).

Target Parameter	Source of Variance	Quadratic Sum	Freedom	Variance	*F*	Significant Difference (*p*)
	phenolic resin	214.111	3	71.37	17.613	0.002
	silica aerogel	47.786	3	15.929	3.931	0.072
pore (>10 μm)	H_2_O_2_	87.57	3	29.19	7.203	0.021
	error	24.313	6	4.052		
	summation	8028.179	16			

**Table 10:** Variance analysis of pore (1–10 μm).

Target Parameter	Source of Variance	Quadratic Sum	Freedom	Variance	*F*	Significant Difference (*p*)
	phenolic resin	418.37	3	139.457	6.705	0.024
	silica aerogel	62.337	3	20.779	0.999	0.455
pore (1–10 μm)	H_2_O_2_	385.376	3	128.459	6.177	0.029
	error	124.785	6	20.797		
	summation	7550.992	16			

**Table 11:** Variance analysis of pore (100 nm–1 μm).

Target Parameter	Source of Variance	Quadratic Sum	Freedom	Variance	*F*	Significant Difference (*p*)
	phenolic resin	2.747	3	0.916	0.113	0.95
	silica aerogel	38.976	3	12.992	1.598	0.286
pore (100 nm–1 μm)	H_2_O_2_	35.468	3	11.823	1.454	0.318
	error	48.776	6	8.129		
	summation	505.33	16			

**Table 12:** Variance analysis of pore (<100 nm).

Target Parameter	Source of Variance	Quadratic Sum	Freedom	Variance	*F*	Significant Difference (*p*)
	phenolic resin	17.258	3	5.753	14.587	0.004
	silica aerogel	5.094	3	1.698	4.306	0.061
pore (<100 nm)	H_2_O_2_	2.046	3	0.682	1.729	0.26
	error	2.366	6	0.394		
	summation	88.741	16			

**Table 13:** Average deviation of the various thermal conduction models.

Thermal Conduction Model	Average Deviation (W/m·K)
Russell model	0.059
Parallel model	0.055
Series model	0.203
Maxwell–Euchen model	0.038
EMT model	0.047
Cheng–Vachon model	0.493

## Data Availability

Not applicable. No data have been created.
